# Herpes Zoster Infection Presenting as Aseptic Meningitis and Dermatomal Rash in Immunocompetent Adult

**DOI:** 10.1155/2020/8571958

**Published:** 2020-01-11

**Authors:** Suyash Dawadi, Sudesh Lamsal, Bhupendra Shah

**Affiliations:** ^1^B.P. Koirala Institute of Health Sciences, Dharan, Nepal; ^2^Department of Internal Medicine, B.P. Koirala Institute of Health Sciences, Dharan, Nepal

## Abstract

Herpes zoster is a localized, painful, and vesicular rash involving one or adjacent dermatomes caused by varicella-zoster virus reactivation. Herpes zoster presenting as aseptic meningitis is prevalent among elderly population and people with immunocompromised status. However, it is a rare phenomenon in the young immunocompetent adult; hence, we are reporting a case of a herpes zoster infection presenting as aseptic meningitis and dermatological manifestation in a 19-year-old immunocompetent male.

## 1. Introduction

Herpes zoster (HZ) is a localized, painful, and vesicular rash involving one or adjacent dermatomes caused by varicella-zoster virus (VZV) reactivation. The incidence of herpes zoster increases with age or immunosuppression [1]. Varicella-zoster virus-specific cell-mediated immunity (CMI) is required to halt the virus reactivation. During young adulthood, VZV-specific CMI is robust which explains the infrequent occurrence of HZ in this age group. Individuals with decreased cell-mediated immunity resulting from carcinoma, radiation therapy, chemotherapy, or human immunodeficiency virus (HIV) infection are at greater risk for reactivation of latent VZV [2]. Herpes zoster is uncommon in healthy immunocompetent individuals; hence, we report a case of herpes zoster infection presenting as aseptic meningitis and dermatomal rash in the immunocompetent adult.

## 2. Case Background

**Figure 1:**
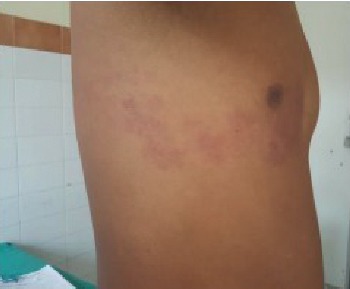
Erythematous vesicles along the right thoracic dermatome of the patient.

A 19-year-old farmer by occupation presented to the emergency room with complaints of a headache for 2 days and vesicular rashes over the right side of the chest for 1 day. The headache was acute in onset, bifrontal, continuous, and throbbing in nature. It was aggravated on the supine position and slightly relieved in the sitting position. The headache was associated with photophobia and multiple episodes of nonprojectile and nonbilious vomiting. The patient did not have any history of fever, altered mental status, altered sleep-wake cycle, loss of consciousness, or any abnormal body movement. The patient complained of painful erythematous lesions on the right side of the chest. The lesions were initially small and later on progressed to become large vesicles. The patient did not have any history of chickenpox in childhood or recent exposure to it. There was no history of any diabetes, immunocompromised status, cardiac diseases, pulmonary diseases or cancer, and immunosuppressive intake. The patient was not on any kind of long-term medication. On general physical examination, the patient was conscious, cooperative, and well oriented to time, place, and person. The patient's blood pressure was 120/80 mmHg, pulse rate was 80 beats per minute, respiratory rate was 18 cycles per minute, and temperature was 98°F. The patient had no pallor, icterus, lymph node enlargement, cyanosis, or clubbing. On systematic examination, the patient had neck stiffness and positive Kernig's sign. The rest of the CNS and systematic examinations were normal. No abnormalities were detected on other systemic examinations. All baseline laboratory parameters were normal except cerebrospinal fluid analysis which showed lymphocytic pleocytosis suggestive of meningitis as illustrated in Table 1. Computed tomography of the head was normal. We admitted the patient with the provisional diagnosis of meningitis and treated with intravenous ceftriaxone 2 gram twice daily and intravenous acyclovir 500 mg thrice daily. On the 2nd day of admission, the patient had a complaint of multiple vesicular, erythematous, and tender lesions on the right side of the chest as depicted in Figure 1. The final diagnosis of herpes zoster infection with aseptic meningitis and dermatome rash was made. We stopped ceftriaxone and continued acyclovir. There was a decrease in headache and vomiting on the 4th day of hospital admission. We discharged on acyclovir 800 mg 5 times a day orally. The patient was followed-up in 3 weeks of hospital discharge without any neurological sequelae.

## 3. Case Discussion

We diagnosed our patient as a case of herpes zoster infection presenting as aseptic meningitis and dermatomal rash. The patient had unilateral single-thoracic dermatomal-distributed painful vesicular skin rash characteristic of herpes zoster rash. Aseptic meningitis was diagnosed as the patient had symptoms of meningeal irritation, lymphocytic pleocytosis, and culture-negative in cerebrospinal fluid and no other explainable noninfectious etiology. However, the serology test for the varicella-zoster virus was not performed due to the unavailability of the test in the hospital.

Herpes zoster is common in the elderly population; as with aging, there is a decline in cell-mediated immunity [3]. The mean age of herpes zoster reactivation was 52 years as reported by Takesima et al. [4]. However, our patient was a young adult with herpes zoster infection presenting as aseptic meningitis and dermatomal rash that make his presentation unique. A similar case report of herpes zoster in the young adult was reported by Kangath et al. [5]. Most of the patients with herpes zoster commonly occurred in the immunocompromised people as reviewed by the Bollea-Garlatti et al. [6]; however, our patient had negative workup for immunocompromised status. Only a few cases with herpes zoster presenting as aseptic meningitis in immunocompetent were reported in the medical literature [7]. Aseptic meningitis prior to vesicular eruption was notable presentation in our patient as Takeshima et al. reported only two of 11 cases had herpes zoster meningitis prior to the dermatological lesion [4]. Similar to our case, herpes zoster aseptic meningitis preceding dermatological rash was reported by Kangath et al. [5].

We treated our patient with intravenous acyclovir 500 mg thrice a day for seven days and oral acyclovir for the next two weeks. The patient had an excellent response to the treatment. Similarly, Kangath et al. reported that the patient with disseminated herpes had a good response with IV acyclovir 500 mg three times a day for seven days and transition to valacyclovir for two additional weeks [5]. We did not prescribe valacyclovir due to its unavailability in the local market. Our patient was discharged after 7 days of hospital stay and followed-up in OPD after 2 weeks and without neurological sequel. We could not perform the polymerase chain reaction of CSF to identify the virus due to the unavailability of the facility in our center.

## 4. Conclusion

Examining the dermatological lesion in a patient with aseptic meningitis may be helpful in diagnosing herpes zoster rarely. Even though herpes zoster infection is common in the elderly, immunecompromised, and patients with comorbidities, it is also possible in a young immunocompetent individual. Early diagnosis of herpes zoster infection in a patient with aseptic meningitis and its prompt treatment with acyclovir often have a good clinical outcome.

## Figures and Tables

**Table 1:** Baseline investigation reports of the patient.

Laboratory parameters	Value	Reference range
Haemoglobin (gm/dl)	16.9	11–16
Haemotocrit (%)	52.7	36–48
TLC (cell/mm^3^)	5400	4000–11000
Platelet (cell/mm^3^)	240000	150000–400000
RBS (mg/dl)	105	<140
HIV antibody	Negative	
CSF analysis		
TLC (cell/mm^3^)	150	<10
Neutrophil (%)	35	
Lymphocyte (%)	65	
RBC (cell/mm^3^)	130	
Glucose (mg/dl)	63	40–65
Protein (mg/dl)	16	15–45
ADA (U/litre)	5	<7.0

TLC: total leucocyte count; RBS: random blood sugar; mg/dl: milligram deciliter; HIV: human immunodeficiency virus; RBC- red blood cell; ADA: adenosine deaminase.
